# Universiti Sains Malaysia Specialist Hospital Leads in Brain Health: Clinical Neurosciences Mind, Psychological Sciences and Psychiatry

**DOI:** 10.21315/mjms-04-2025-253

**Published:** 2025-04-30

**Authors:** Muhammad Ihfaz Ismail, Song Yee Ang, Diana Noma Fitzrol, Zaitun Zakaria

**Affiliations:** 1 Department of Neurosciences and Brain Behaviour Cluster, School of Medical Sciences, Universiti Sains Malaysia, Health Campus, Kelantan, Malaysia; 2 Universiti Sains Malaysia Specialist Hospital, Universiti Sains Malaysia, Health Campus, Kelantan, Malaysia

Dear Editor,

We read with interest the article in the *Malaysian Journal of Medical Sciences* (MJMS) (1) written in 2015 regarding brain health and the advancement being made in our medical community since the establishment of clinical neurosciences, mind, cognitive and psychological science in 1999.

Ten years later, we celebrate the 25-year anniversary of the first vagus nerve stimulation implant performed at Hospital Pakar Universiti Sains Malaysia by Professor Dato’ Dr. Jafri Malin Abdullah which reflects the significant strides we have made in neurosurgery and neurology. This milestone, marked by the implantation of generator model 100C (Serial Number 16806) on 30 September 2000, is a testament to our dedication to innovative Professor Dato’ Dr. Jafri Malin Abdullah continues to make significant contributions to neurosurgery and neurology. Thereafter, this vagus nerve stimulation implantation continued by Professor Zamzuri Idris and Dr. Ang Song Yee.

Professor Dato’ Dr. Jafri Malin Abdullah has shown extensive dedication to the field and has recently been acknowledged with several prestigious awards; Research Excellence Award by the Ministry of Higher Education (MOHE) – Elsevier on 28 November 2024 under the category of National Societal Impact 2024 and he was also listed thrice as top 2% in the World in their respective field by Stanford University. Beside this, he was also appointed as a Steering Committee Member of the Brain Capital Alliance and Brain Economy Hub, and the International Neuro Climate Working Group and selected as the Representative for the Global Brain Consortium Committee Chapter for Southeast Asia. Furthermore, he continued as the Chairman of Brain Behaviour Cluster of Universiti Sains Malaysia from 2017 until 2025. This consistent recognition reflects his sustained commitment to excellence and innovation within the field (Figure 2–6).

Notably, the work of Professor Dato’ Dr. Jafri Malin Abdullah and Dr. Muhammad Ihfaz Ismail in performing the first Bonnet bypass—a long interposition vascular graft bypass used for extracranial to intracranial revascularisation— provides an important alternative for patients with large vessel occlusions who do not have a suitable ipsilateral donor or in whom the ipsilateral donor must be sacrificed (2). This is particularly significant in Southeast Asia for patients who cannot afford endovascular treatment (Figure 7).[Fig f1-17mjms3202_le]

Furthermore, Bonnet emergency bypass was successfully performed on 3 November 2024, demonstrating our team’s capability to respond to critical cases effectively (Figure 7). Our extensive provision of STA-MCA bypasses for chronic occlusion or Moya-moya disease to regions such as Sabah, Ipoh, Johor Bahru and Sungai Buloh showcases our growing capabilities in addressing complex neurological conditions. As of 5 April 2025, we have marked the 38th case of STA-MCA bypass since the first bypass case started at Hospital Pakar Universiti Sains Malaysia since 20 May 2023.

Our centre offers a unique service of carbon dioxide (CO_2_) challenge test as a routine pre-operative investigation before embark on bypass surgery. On top of that, our centre also routinely performs transcranial doppler after aneurysm surgery to detect early evidence of vasospasm and if there is evidence of vasospasm, we routinely give stellate ganglion block to treat vasospasm and we also routinely use ultrasound orbital to check for optic nerve sheath diameter for those patients who are not on external ventricular drainage for intracranial pressure (ICP) monitoring.

Additionally, Dr. Lee Foo Chiang (neurosurgeon) from Sunway Hospital, who began providing deep brain stimulation (DBS) services in 2003, along with Dr. Chee Chee Pin (neurosurgeon) and Dr. Lee Moon Keen (neurologist) (Figure 8–9), have significantly supported our comprehensive approach to treating patients with movement disorders and this had lead to future generations of neuromodulation neurospecialist headed by Professor Dato’ Dr. Abdul Rahman Izaini Ghani and team who also does complex spine surgery (Figure 10).

Besides, our Head of Department, Professor Zamzuri Idris possesses exceptional skills in epilepsy surgery and skull base surgery, and he has performed awake cranial surgery, showcasing his expertise in complex procedures. His ability to navigate complex surgical challenges allows him to provide innovative treatment options for patients with difficult-totreat conditions, thereby offering hope where it was previously lacking. Recently, on 27 January 2025, Hospital Pakar Universiti Sains Malaysia performed the first vagal nerve stimulation implantation of a new model of SENTIVA in Southeast Asia (Figure 10).

Furthermore, Professor Zamzuri Idris, together with Dr. Azim Patar, our neuroscientist, have established a stem cell project in the 13th Malaysia Plan, which is Malaysia’s roadmap for the period of 2026–2030. In addition, he is actively involved in many scientific research projects. His latest publication, entitled “Magnetoencephalography Brain Spectral Biomarkers of Alzhiemer’s Disease: A Promising Approach Demonstrated with BioFIND Dataset,” was a cutting-edge paper which won the overall best paper award (Figure 11).

Moreover, Professor Dato’ Dr. Abdul Rahman Izaini Ghani has been instrumental in advancing DBS techniques. His recent accomplishment of performing the 373rd case of DBS for the entire nation on 5 April 2024 highlights not only his exceptional surgical skills but also his commitment to improving the quality of life for patients with movement disorders across Malaysia. Besides, patients who are workers insured by the Pertubuhan Keselamatan Sosial (PERKESO) receive full support for this deep brain stimulation (DBS) procedure. His expertise extends beyond DBS service delivery at HPUSM, as he also performs surgeries at Hospital Umum Sarawak, Hospital Seremban, and Hospital Sungai Buloh.

We hope Hospital Pakar Universiti Sains Malaysia (HPUSM) will soon be a spinal cord stimulation referral centre. We congratulate Professor Dato’ Dr. Abdul Rahman Izaini Ghani, who was elected as the Hospital Director of HPUSM on 1 January 2025.

Furthermore, Dr. Muhammad Ihfaz Ismail has received a one-year fellowship in Pain and Spine Intervention and a one-year fellowship in Spine Endoscopy, enabling him to provide minimally invasive awake endoscopic spine decompression for spinal stenosis since 30 April 2024. He also provides various interventional pain management techniques, including dorsal root ganglia blocks, medial branch blocks, and piriformis blocks, thereby expanding the range of treatment options for patients suffering from spinal conditions.

Dr. Diana has developed a keen interest in peripheral nerve surgery and has participated in numerous peripheral nerve surgeries alongside the orthopaedic team, further enhancing our surgical capabilities. She recently performed the posterior tibial neurectomy under electromyography (EMG) intra-operative monitoring on 6 March 2025. She is also in charge of brain tumour cases treated by intraoperative radiotherapy since its establishment more than 10 years ago. From January 2024 until April 2025, 15 cases have been managed by intra-operative radiotherapy (IORT).

The commitment to further education and training is evident as our team participates in advanced learning opportunities globally. Dr. Muhammad Ihfaz Ismail will be heading to Hyogo Medical University for further training in endovascular interventions, including thrombectomy, embolisation, stenting, and coiling. Dr. Diana went to Japan for endoscopic transsphenoidal surgery and recently trained in Bangkok for spinal cord stimulation, Dr. Zaitun gained experience in paediatric surgery in Dublin, and Dr. Ang also went to Japan for endoscopic transsphenoidal surgery and has upcoming training in China for DBS representing a proactive approach to skill enhancement.

In addition, Associate Professor Dr. Sanihah Abdul Halim, our neurologist, is working hand in hand with Dr. Nasibah Mohamad and her team of interventional radiologists for advanced stroke management. Besides, Associate Professor Dr. Sanihah also actively collaborates with Dr. Mohd Faizal Mohd Zulkifly, our Clinical Psychologist and Associate Professor Dr. Asrenee Abd Razak, Consultant Psychiatrist for a combined clinic for transcranial magnetic stimulation for various indications, especially neurocognitive disorders and depression.

To ensure that we continue to provide exceptional care and remain at the forefront of neurosurgical advancements, it is imperative that we prioritise and invest in expanding our human resource capabilities. Increased training, mentorship, and recruitment will ultimately enhance our ability to serve patients effectively and improve brain health outcomes across Malaysia

Together, let us advocate for the development of a robust workforce dedicated to brain health, leveraging the skills and expertise necessary to meet the evolving needs of our society.

Besides, it is our commitment that department’s manpower continuously produce students with various backgrounds via our training programmes for the 2024/2025 batch to meet the nation’s needs. Programmes such as Master of Neurosurgery (Figure 13), Master of Cognitive Neurosciences (Figure 14), and Master and Doctorate in Clinical Psychology (Figure 15) will contribute to Malaysia’s neurological and mental health services.

## Figures and Tables

**Figure 1 f1-17mjms3202_le:**
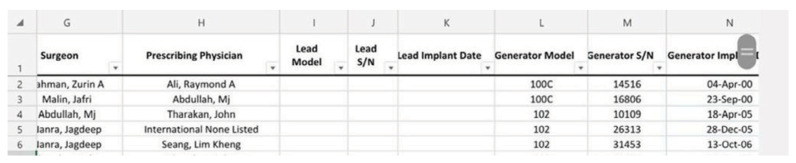
The 25th anniversary of the first vagus nerve stimulation implant at Hospital Pakar Universiti Sains Malaysia, performed by Professor Dato’ Dr. Jafri Malin Abdullah. The generator model 100C with serial number 16806 was implanted on 23 September 2000

**Figure 2:**
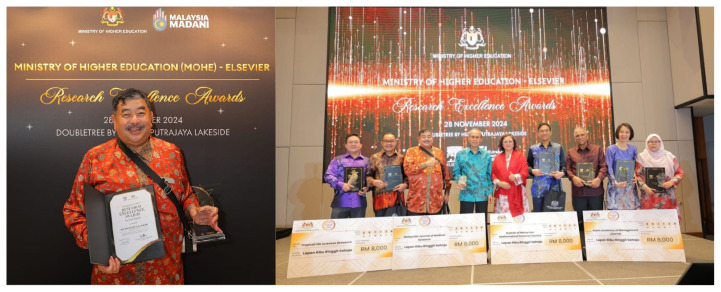
Research Excellence Award by Ministry of Higher Education (MOHE) – Elsevier on 28 November 2024

**Figure 3:**
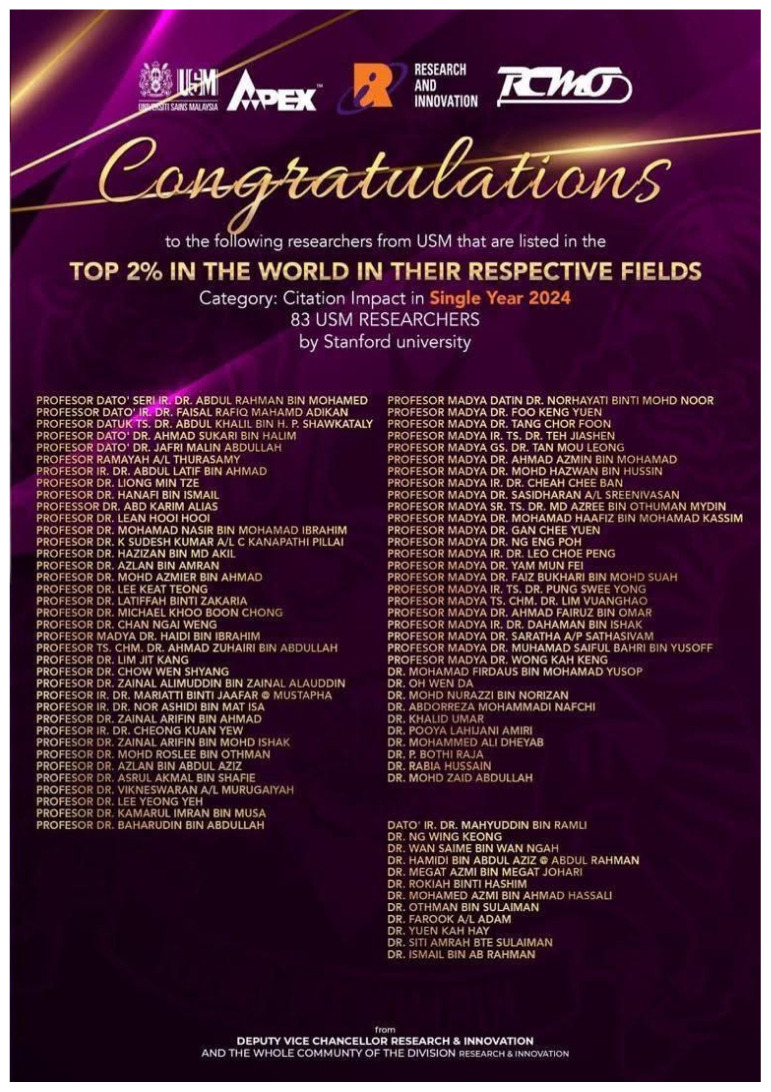
Professor Dato’ Dr. Jafri Malin Abdullah was listed as top 2% in the World in their respective field by Stanford University three times in a row

**Figure 4:**
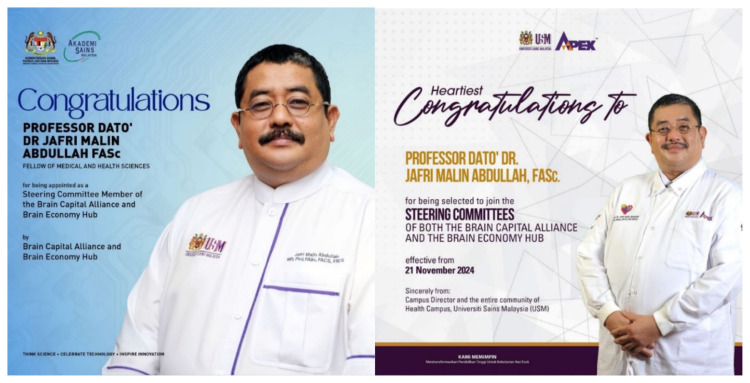
Professor Dato’ Dr. Jafri Malin Abdullah was appointed as a Steering Committee Member of the Brain Capital Alliance, Brain Economy Hub by Academy of Science Malaysia

**Figure 5:**
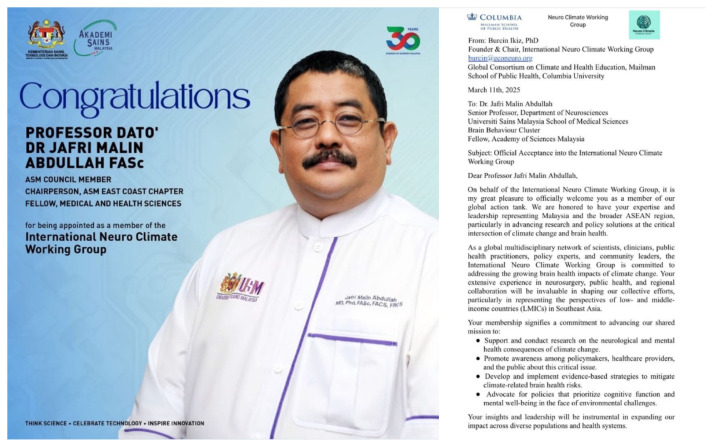
Appointed as a member of the International Neuro Climate Working Group, USA

**Figure 6:**
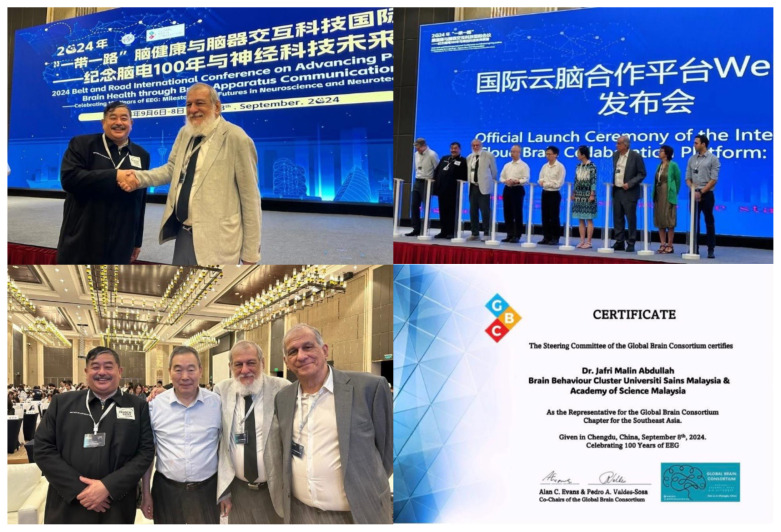
Professor Dato’ Dr. Jafri Malin Abdullah attended the 2024 Belt and Road International Conference on Advancing Population Brain Health through Brain-Apparatus Communication on 6–8 September 2024. In the same event, he was also appointed as Representative for the Global Brain Consortium Committee Chapter for Southeast Asia

**Figure 7:**
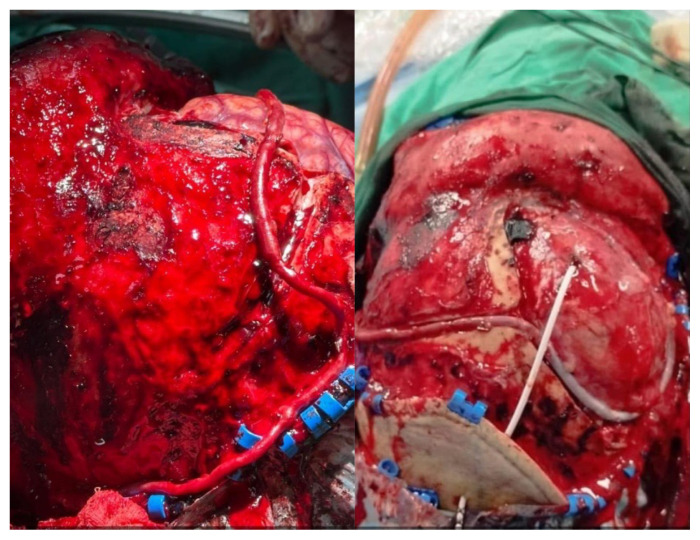
A long saphenous vein was used as a graft to connect from left external carotid artery to right middle cerebral artery (M4 segment)

**Figure 8:**
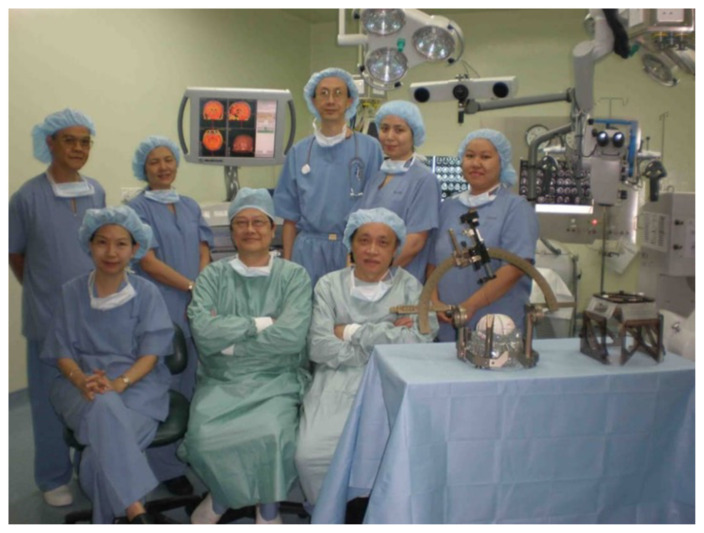
Dr. Lee Foo Chiang (neurosurgeon), front row centre; Dr. Chee Chee Pin (neurosurgeon), front row right; and Dr. Lee Moon Keen (neurologist), front row left

**Figure 9:**
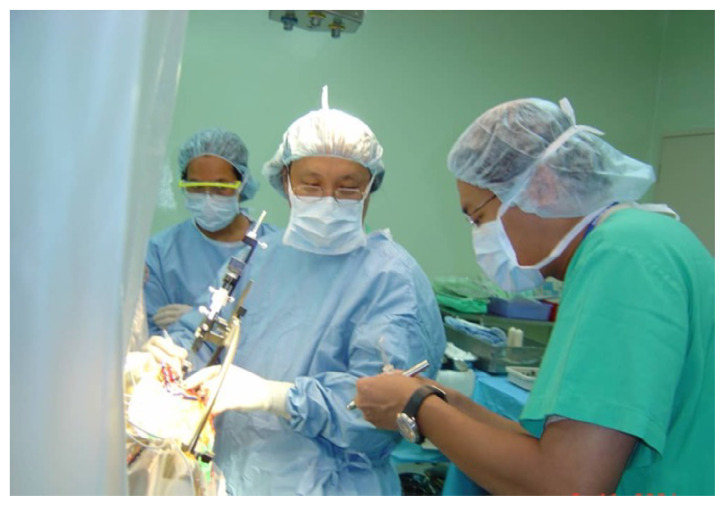
DBS at Sunway Hospital in 2003 by Dr. Lee Foo Chiang

**Figure 10:**
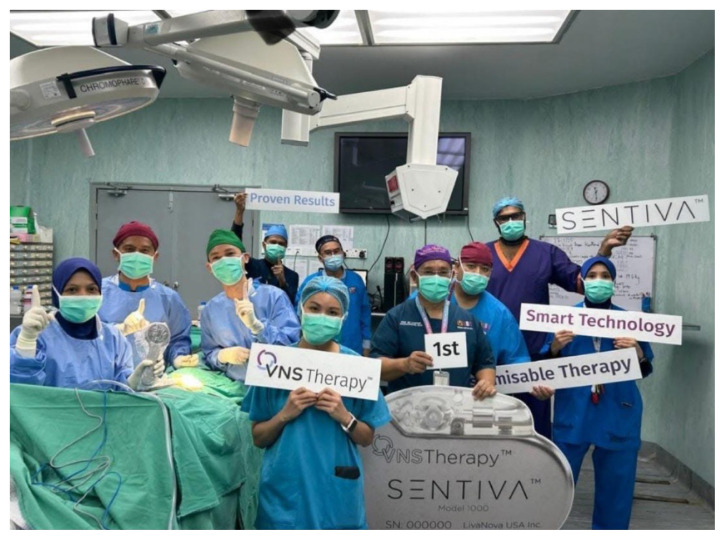
Professor Zamzuri Idris led the vagal nerve stimulation implantation on 27 January 2025

**Figure 11:**
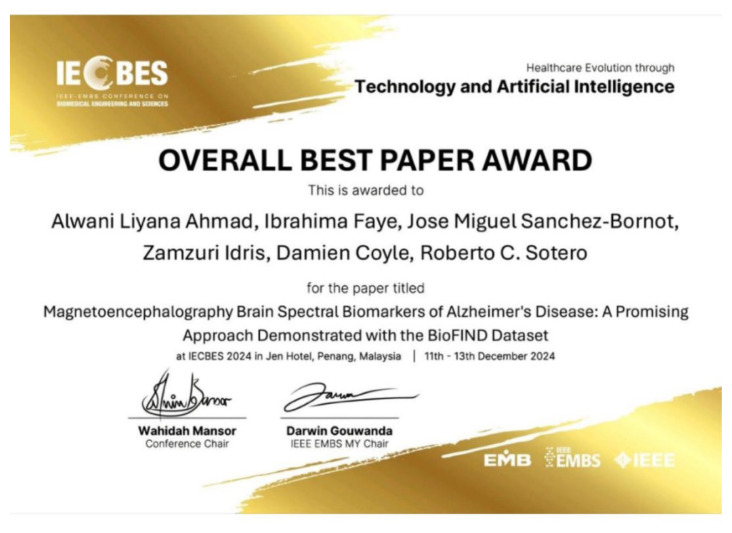
Overall best paper award

**Figure 12:**
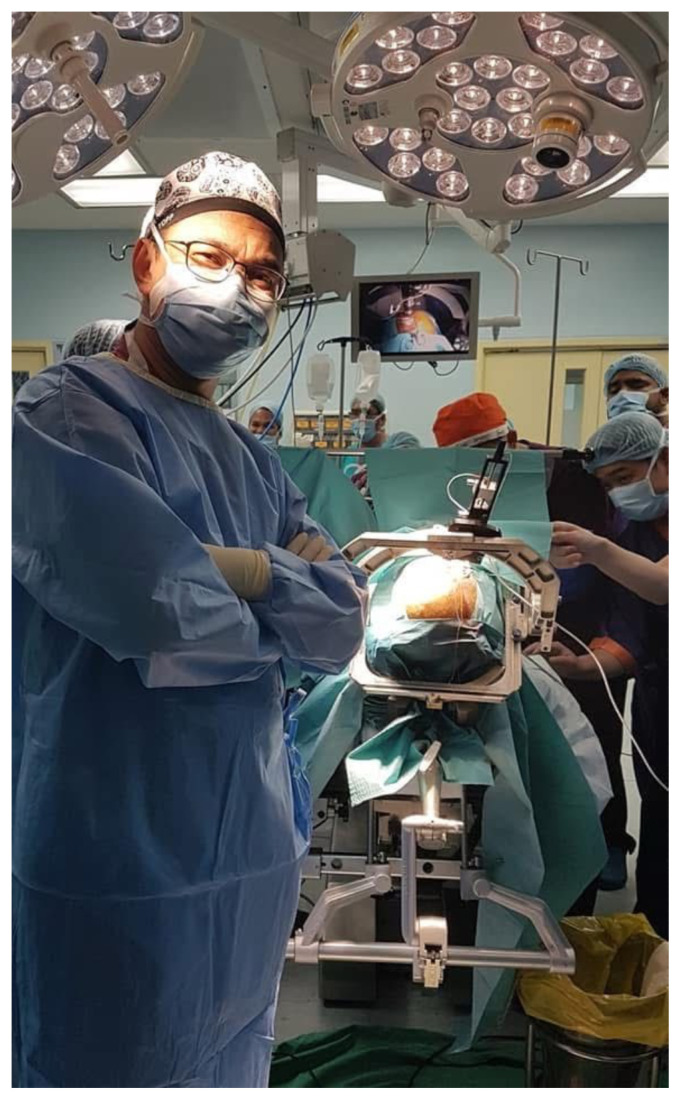
Professor Dato’ Dr. Abdul Rahman Izaini performed one of the DBS cases for his Parkinson’s patient

**Figure 13:**
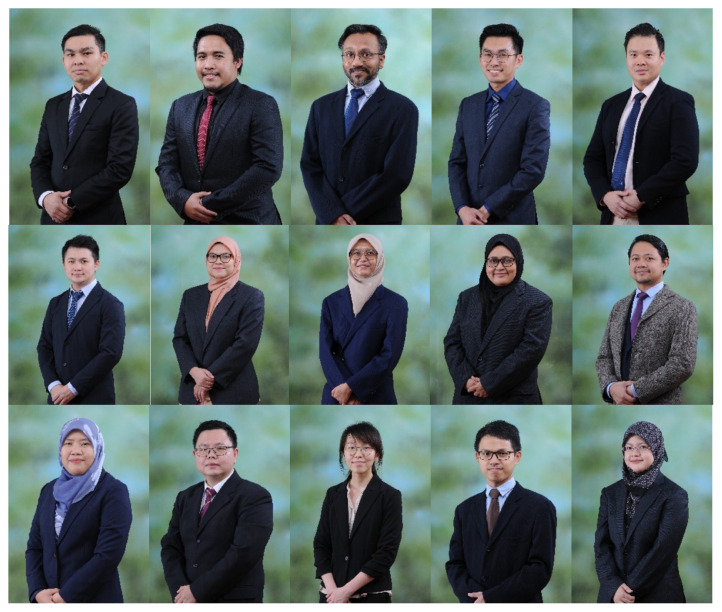
MMED Neurosurgery (intake 2024/2025), from left to right: Dr. Ahmad Hakimy Ahmad Puahad, Dr. Che Ku Ashraf Helmi Che Ku Mazuan, Dr. Devalagan Muthalagan, Dr. Kung Khai Shien, Dr. Law Puong You, Dr. Liang Kevin, Dr. Nadiah Nasuha Zolkaflee, Dr. Norhidayah Jamil, Dr. Nur Amirah Fazira Malzuki, Dr. Shah Ozair Shaharuddin, Dr. Sharalini Zainuddin, Dr. Tan Beng Ping, Dr. Tan Kia Hooi, Dr. The Ping An, Dr. Wan Noor Farzana Wan Mohd Salleh

**Figure 14:**
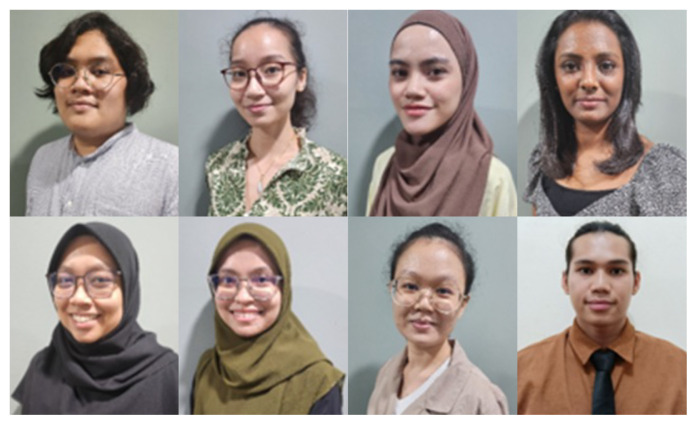
Master of Cognitive Neurosciences student. From left to right, 12th batch, intake March 2024 (7 students): Abang Muhammad Diniy Abang Madehan, Lily Qistina Abdul Manan, Nurul Amira Natashya Halim, Esther Sager Albert, Nur Munirah Mohd Taufik, Khadijah Abdullah Md Harashid, Lim Hooi Ping; 13th batch, intake October 2024: Muhammad Ghazalie Mohamad Sabri

**Figure 15:**
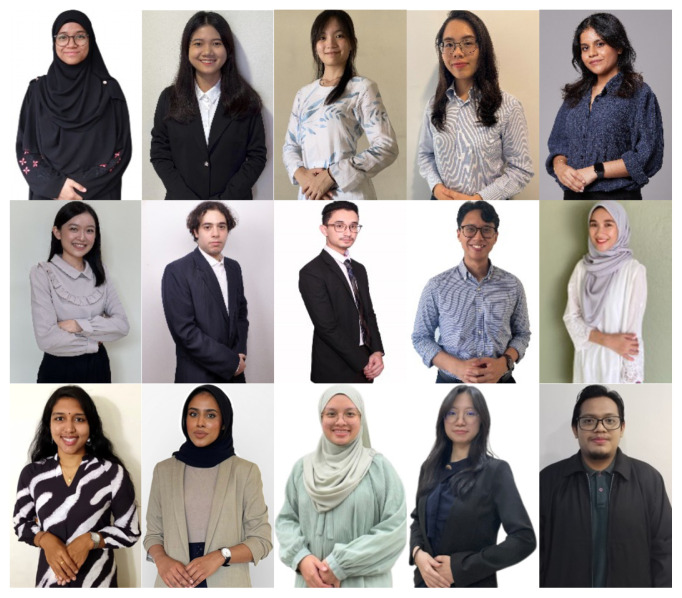
Cohort 7 USM-UPSI Integrated Psychology Programme. From left to right, Master in Clinical Psychology (14 students): Aisyah Murshidah, Chey Yi Ting, Crystal Ng Kah Yan, Ee Quan Lim, Khishantini Keasavan, Lim Chu Yeng, Mohammaed Alasaad, Muhammad Amin Mohd Hamzah, Presley Jairom, Siti Syahmina Shuhainor, Umassree Murugesan, Umul Jasrina Sied Abd Jalil, Wan Nur Najwa Nasuha Wan Ata, Yip Huiye; Doctorate in Clinical Psychology: Mohamed Nasrun Mohamed Zikrillah
